# Studying the laws of software evolution in a long-lived FLOSS project

**DOI:** 10.1002/smr.1615

**Published:** 2013-10-14

**Authors:** Jesus M Gonzalez-Barahona, Gregorio Robles, Israel Herraiz, Felipe Ortega

**Affiliations:** 1 GSyC/LibreSoft, Universidad Rey Juan CarlosFuenlabrada, Spain; 2 Universidad Politécnica de MadridMadrid, Spain

**Keywords:** free software, open source software, software evolution, source code management system, mining software repositories

## Abstract

Some free, open-source software projects have been around for quite a long time, the longest living ones dating from the early 1980s. For some of them, detailed information about their evolution is available in source code management systems tracking all their code changes for periods of more than 15 years. This paper examines in detail the evolution of one of such projects, glibc, with the main aim of understanding how it evolved and how it matched Lehman's laws of software evolution. As a result, we have developed a methodology for studying the evolution of such long-lived projects based on the information in their source code management repository, described in detail several aspects of the history of glibc, including some activity and size metrics, and found how some of the laws of software evolution may not hold in this case. © 2013 The Authors. Journal of Software: Evolution and Process published by John Wiley & Sons Ltd.

## 1. INTRODUCTION

Large, long-lived software projects are difficult to study. Until some years ago, having access to one of them was very difficult and time-consuming. In addition, reliable information about them has to be available for all the period under study. If available, it still has to be found, extracted, and analyzed to produce meaningful time series and other data artifacts that can be studied to gain knowledge about its evolution.

This situation changed during the last years with the public availability of mature free, open-source software (FLOSS) projects, some of which now feature more than 15 or even 20 years of history. Many have been supported by source code management (SCM) systems over a large fraction of their life, and the corresponding repositories have been maintained carefully enough to still have much of the historical information available, with enough reliability and detail to allow for the derivation of significant conclusions.

In this paper, we study one of those long-lived FLOSS projects, glibc, with over 20 years of history in its SCM repository. Evolution in this kind of large, long-lived projects has many edges: all of them should be considered to understand the whole story. Our purpose is to analyze this wealth of information using a repeatable methodology that allows for the analysis of several aspects of the evolution of the project. In the process, we have also identified several problems and shortcomings of the available data that have to be taken into account for such analysis. More in particular, the main goal of the study can be summarized as follows:

To do a bottom-up analysis of the evolution of the software development project, on the basis of the information available in its SCM system, in the context of the laws of software evolution.

As it is mentioned in this statement of principles, we have used the framework provided by the laws of software evolution 1. This has allowed us to focus on a certain set of parameters, consistent with those considered by the laws; while at the same time, we have been able to assess the validity of some of them in the specific case of our subject of study. Instead of coming from the laws and then trying to decide which parameters to use for the analysis, we have started with the parameters that can be extracted from the SCM repository and have found how to use them to apply the laws.

A basic assumption of our study is, therefore, that the characterization of the evolution of the project can be carried out by analyzing the data available in its SCM repository. Our methodology produces the parameters needed to check the laws using that data, defining exactly which information is to be analyzed, which processes have to be followed for producing the relevant parameters from it, and how they are to be interpreted in the context of the laws of software evolution.

During the design and testing of the methodology in the specific case of glibc, we decided which of the laws were useful in our case, and at the same time, we overcome the vagueness of some of them. For the former, we focused on those that deal with parameters that can be extracted from the meta-information in the SCM repository. For the latter, we considered what can be inferred from the SCM repository in the context of the previous studies in software evolution.

To produce the final methodology presented in this paper, we followed an iterative process in which a tentative methodology was followed until some new issue was discovered, which allowed for its refinement and further testing. The resulting methodology can be summarized in the following steps:

1. Data retrieval. Identification of the SCM repository for the project and retrieval of the meta-information in it.
2. eneral inspection and validation. General inspection of the resulting data, looking for sources of error and specific events and patterns (such as migrations between SCM systems or long-term patterns of use) that could influence the results.
3. Overall activity. Calculation of parameters related to the overall activity, which capture information about the total activity and the size of the development team over time, allowing for the identification of phases in the history of the project.
4. Development activity. Identification of the development branch and calculation of parameters related to its activity, which capture information that can be interpreted in the context of the laws of software evolution. This is as opposed to, for example, stabilization activity that happens in release branches.
5. Source code evolution. Calculation of parameters related to the size and complexity of the source code as it evolves in the development branch, also suitable for interpretation in the context of the laws.

As a result, this methodology proposes precise mechanisms for interpreting the actual meaning of some of the laws of software evolution, therefore, allowing for their validation (or not) in the case of specific projects. When applying these mechanisms to the SCM repository used by glibc, we were able to assess their validity for this specific case.

The structure of the rest of this paper reflects the schema described in this introduction. It starts with a brief introduction to the laws of software evolution (in the next section), followed by a general description of the glibc project and the characterization of the data retrieval step of the methodology (Section 3). Then, the next steps of the methodology are described: general inspection of the retrieved data (Section 4), characterization of the overall activity (Section 5), of the development activity (Section 6), and of the source code evolution (Section 7). In each of these cases, the application of the corresponding step to the glibc case is included in the same section. After that, Section 8 discusses the findings related to the laws of software evolution in the case of glibc, followed by an analysis of the threats to validity (Section 9), and a review of previous research (Section 10). The paper ends with a section on conclusions and further work and some information about the reproducibility elements and methodological details that are offered in a companion website.

## 2. THE LAWS OF SOFTWARE EVOLUTION

Studies on software evolution started in the late 1960s with the pioneering work of M. M. Lehman 2. This work led to the definition of the so-called *laws of software evolution*, whose latest form was published in 1996 1 (Table 1). For about a decade, the laws of software evolution were thought to be valid for large software projects, considering ‘large’ as a property of the development project as a whole, not only of the code size. Those large projects were therefore considered to be developed by large teams organized in several managerial levels, with some history available to be studied, and with a feedback-driven development process thanks to its users.

**Table 1:** Current formulation of the laws of software evolution.

I	Law of continuing change
	An *E*-type system must be continually adapted, else it becomes progressively less satisfactory in use.
II	Law of increasing complexity
	As an *E*-type is changed, its complexity increases and becomes more difficult to evolve unless work is performed to maintain or to reduce the complexity.
III	Law of self-regulation
	Global *E*-type system evolution is feedback regulated.
IV	Law of conservation of organizational stability
	The work rate of an organization evolving an *E*-type software system tends to be constant over the operational lifetime of that system or phases of that lifetime.
V	Law of conservation of familiarity
	In general, the incremental growth (growth rate trend) of *E*-type systems is constrained by the need to maintain familiarity.
VI	Law of continuing growth
	The functional capability of *E*-type systems must be continually enhanced to maintain user satisfaction over system lifetime.
VII	Law of declining quality
	Unless rigorously adapted and evolved to take into account changes in the operational environment, the quality of an *E*-type system will appear to be declining.
VIII	Law of feedback system
	*E*-type evolution processes are multilevel, multiloop, and multiagent feedback systems.

After the initial formulation of the laws, Lehman substituted in 1980 the condition of ‘large projects’ by a more specific classification, the SPE scheme 3, which defines three types of programs:

- *S*-type (specified) programs are derivable from a static specification and can be formally proven correct or not.
- *P*-type (problem solving) programs attempt to solve problems that can be formally formulated, but this formal approach is not affordable from a computational point of view. Therefore, the program must be based on heuristics or approximations to the theoretical problem.
- *E*-type (evolutionary) programs are reflections of human processes or of a part of the real world. These programs attempt to solve an activity that somehow involves people or the real world.

*S*-type programs do not evolve, because once the program is written, it is either correct or not (i.e., the program fulfills the specification or not).

*E*-type software shows evolutionary behavior, as it is supposed to be a model of a part of the real world. Because the real world changes, *E*-type programs must evolve to adapt to those changes. Changes of the real world may appear as changes in the specification, demand of new features, discovery of defects after release, and so on. In other words, specifications that model a part of the real world, particularly if it involves human processes, will need to be refined or adapted in the future.

*P*-type is an intermediate case between the *S*-type and *E*-type. In this case, the specification cannot be completely defined before the program is implemented. More recently 4, its definition has been adapted to refer to *paradigm-based* software. A paradigm is a specification of the real world that can change as knowledge or practice evolves. With this new definition, *P*-type specifications can model non-static problems, with changes in the paradigms being the source of changes in the specification.

The laws exclusively describe *E*-type software and do not mention either *S*-type or *P*-type. *S*-type software does not evolve, so it is clear why it is not included. However, *P*-type software does evolve. The reason for not considering *P*-type software is because of one of the cornerstone concepts of Lehman's theory of software evolution: the driving force of the evolution of software is feedback. Whereas the source of feedback in *E*-type software is stakeholders demanding changes to adapt the program (either to include new features or to solve discovered defects), the changes in *P*-type software are due to the nature of the specification or paradigm it implements. Changes in *P*-type software are not continuous, as they happen from time to time when the paradigm is updated.

## 3. THE GLIBC PROJECT AND THE RETRIEVAL FROM ITS SCM DATA

The first step in the methodology is the identification of the SCM repository corresponding to the project to analyze and the retrieval from the relevant information from it. To provide some background, we also include in this section a brief discussion on the glibc project, which will be our case study for applying the methodology.

### 3.1. The glibc project

The glibc (http://www.gnu.org/software/libc/) is a C standard library released by the Free Software Foundation (FSF). It is used on top of many operating system kernels and hardware systems. Currently, it officially supports the following architectures: x86, Motorola 680x0, DEC Alpha, PowerPC, ETRAX CRIS, s390, and SPARC. It provides the functionality specified in the Single UNIX Specification, POSIX (1c, 1d, and 1j), some of the functionality specified by the ISO C99 and Berkeley Unix (BSD) interfaces, the System V Interface Definition and the X/Open Portability Guide, and some extra functionality that has been demanded in some of the systems where it is used.

The history of glibc is long. It is a part of the GNU project, promoted by the FSF. Already in February 1988, the FSF described it 5 as ‘a nearly complete set of ANSI C library functions’. In January 1992, they commented 6 that glibc ‘contains all of the ANSI C-1989 and POSIX.1-1990 functions, and work is in progress on POSIX.2 and Unix functions (BSD and System V)’. In February of that same year, glibc 1.0 was released. At about that time, it was forked by the developers of the Linux kernel, which maintained it separately for several years, until 1996, when glibc 2.0 was released by the FSF, and it was reincorporated in most Linux-based distributions until today. Since 2001, the development of glibc is overseen by the GNU C Library Steering Committee.

From the point of view of software evolution theory, it could be discussed whether glibc falls into the *P*-type or *E*-type categories. Because it is written mostly to satisfy the specifications of several well-defined interfaces (as mentioned previously), it could be argued that it should be classified as *P*-type. However, the set of specifications and the supported architectures evolved so much over time that it has to be considered as *E*-type, where adaption to unforeseen changes is strictly needed to remain functional and attractive for prospective users. The fact that in 1992, the Linux development team forked it comes in support of this assumption: they were not happy with how the library was evolving at that time, to the point of maintaining their own version. By 1996, however, it had adapted enough to their needs to be adopted back. Therefore, for the purposes of this paper, we will consider that glibc is *E*-type and therefore should be subject to the laws of software evolution.

### 3.2. Data retrieval

The glibc is currently supported by a git SCM system (the official git repository for the project is located at http://git://sourceware.org/git/glibc.git). The study presented in this paper was performed by cloning the git repository and extracting information from it with CVSAnalY2, * storing the resulting data in a MySQL database. That database was later analyzed with the help of the R statistics tool, which produced the graphs found in the next sections. A specific extension was developed for CVSAnalY2 to obtain information about lines added and removed for each commit.

## 4. GENERAL INSPECTION OF THE SOURCE CODE MANAGEMENT INFORMATION

Before starting with the analysis of the data retrieved from the SCM repository, we propose to perform a general inspection of it. This allows for the identification and minimization of several potential sources of error and provides some hints that will be useful when interpreting the results of the analysis.

### 4.1. Methodological issues

The following questions should be answered in this step:

- Can any source of error be found? A first inspection of the SCM repository should be concerned with the correctness of the data. General queries should be performed to identify suspicious situations. Some examples of those situations are wrong commit dates or out-of-order commits. The amount of errors should be evaluated to see how much it may affect the study. If possible, these errors should be corrected. If not, they should be at least highlighted and omitted in the following analysis. This also includes the validation of the data by random cross-inspection of the repository and the information in the database.
- Has the project migrated between repositories? During the lifetime of long-lived projects, several repositories might have been used. Although the usual practice is to follow a migration procedure that does not lose data, sometimes errors occur that can cause problems. For example, certain comments in commit records may be missing, or some committers may not be properly identified. In addition, changing repositories, as well as the SCM handling them, can add some methodological challenges that need to be addressed 7.
- Has the SCM been used consistently (and constantly)? The existence of a SCM does not imply its use, especially in old projects where the use of SCM was testimonial. In addition, the practices may have changed over time, as the development culture or the tools may have changed.
- Is the history complete? Although at least a part of the changes originated elsewhere end in the official repository at some point, evolution may not only happen in the ‘official’ repository but in many other places. This effect explains how projects can grow quickly during periods of low activity, for example, by receiving code being developed elsewhere.

### 4.2. The glibc source code management repository

The first SCM system that glibc used was CVS. Migration to git happened on May 15, 2009, preserving all version history and most of the meta-information, as was observed by inspection. However, the migration caused that some comments to commits have not been properly dealt with and are missing or partially missing in the git commit records. After some manual inspection of a sample of the meta-information in the git log, other loss of relevant information because of that migration has not been observed. However, and probably because of some maintenance issues in the machine where the CVS service was hosted, there are some anomalies in the dates of the current git log: some commit dates are not consistent with either the history of glibc or the sequence of changes. For example, the oldest commit is dated from ‘Sat May 13 23:00:00 1972’, which is clearly impossible for a project that started in the late 1980s. Some commits have also been observed to be out of order.

However, a semiautomated inspection of commits (including comparison of logical ordering with date ordering) has shown only a very small number of such cases, which means that most of the information in the current git repository seems to be correct. Thanks to the very detailed and accurate Changelog files maintained by the glibc team, we have been able to compare the history as reported by the log of the SCM repository and the history as it is reported by glibc developers, concluding that there are no discrepancies between them, except for some loss of granularity in some large commits, which we explain in more detail in Section 8.

Except for the mentioned anomalous first commit, the commit history starts on ‘Mon Feb 20 02:20:28 1989’ with the comment ‘Initial revision’, which seems to flag the first file put under revision control. For a while, the repository is used seldomly, with only some files being sporadically added to it until January 30, 1991. On that date, the real use of the repository seems to start, when 144 files are added (less that 100 were added in the previous months), and commits start to flow in with continuity. The analysis presented in this paper ends with a commit performed ‘Tue Feb 28 16:37:58 2012’. For the purposes of our study, we will study the evolution between 1991 and 2011, both years included.

From February 1991 on, the most observable discontinuity was caused by the aforementioned migration from CVS to git, with developers changing their commit patterns. Commits are smaller (very large commits have disappeared), and a large fraction of development actions are now performed in branches. The size of changes and the number of changes per commit help to understand the migration from CVS to git: since 2009, large commits have disappeared from the graphs. However, it could be also the case that other factors affect this discontinuity, such as changes in the level of new functionality added.

During the period of study, several parallel branches of development and maintenance have been active in the SCM repository. With a model similar to Feitelson's *perpetual development model* 8, growth and new functionality only occur in the *master* (main) branch, whereas other parallel branches are reserved for bug fixing activities in stable versions. More recently, the repository also contains some developers' personal branches. Therefore, activity in all branches is a proxy of the activity in the project as a whole, including all prerelease and postrelease work in specific branches (which are mainly production-only branches), whereas the main branch (which is mainly the development branch) receives the changes to the ‘base’ code, which is evolving over time, and will therefore the one to be analyzed for evolution studies.

## 5. OVERALL ACTIVITY

Once the information from the SCM repository has been inspected, it is the time of analyzing the overall activity that this information reveals.

### 5.1. Methodological issues

There are two kinds of parameters that we use for characterizing the overall activity:

- Level of activity. The overall level of activity will be a primary source of information for the identification of phases in the project. For characterizing it, we use the number of commits per month. Using this parameter, it is possible to identify different development phases of the project. As it will be shown in the case of glibc, the history of the project can then be better explained after this identification.
- Active development team. The analysis of the development team is performed from three perspectives: committers per month, total committer months, and ratio of commits per committer. The number of committers per month represents the size of the active developer community at any given time. Adding up all the committers for each month gives an estimation of the maximum effort (in person-months) for code-changing tasks in the project. And analyzing the number of commits per committer for each month is a proxy for the mean activity of developers. To obtain these parameters, developers with multiple identities during the periods of study (months in this case) have to be properly identified, to not overestimate the number of developers.

The identification of phases in the development that we include in this step can already be found in the initial works by Lehman 9. In them, the evolution of a program is assumed to happen in two main stages: development and evolution. The software is developed until it is released, then it enters the maintenance stage. However, the laws of software evolution do not assume any particular type of phased lifetime and others could be possible.

The identification of phases has been later integrated more closely with the theory about software evolution as the staged model of software evolution 10, which divides the lifetime of a software project in five stages: initial development, evolution, servicing (no new features, only *patches* to found problems), phase-out (when a new system substituting the old one is released), and close-down (the system is discarded). This model has been shown to match well the usual evolutionary patterns of FLOSS projects 11, with some empirical evidence of the different evolutionary behavior in different phases 12.

### 5.2. Activity in glibc

**Figure 1:**
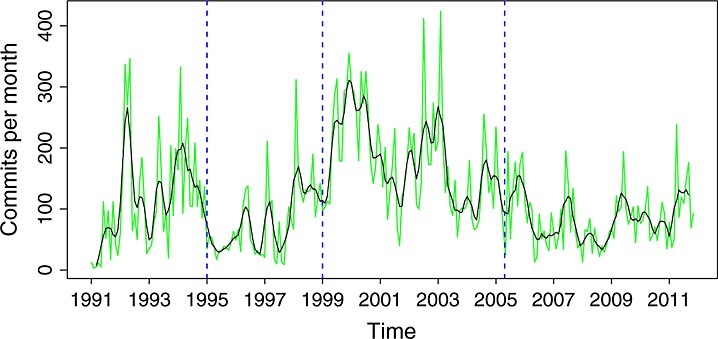
Commits per month in the source code management repository (all branches). In green is the actual data. In black is a smoothed version of it (using a two-sided moving average).

The overall activity in glibc over the whole period of study is shown in Figure 1. From it, four phases of activity can be identified (Table 2, and dashed lines in all time series figures). However, these phases are not uniform. There are clear peaks in 1992, around 2000, and in 2003, among others, and valleys also abound.

**Table 2:** Phases in the activity of the project as a whole. Dates and commits per month are approximate.

Phase	Duration	Activity	Commits/month
A	1991–1994	High	100
B	1995–1998	Low	50
C	1999–2005	Very high	130
D	2006–2011	Moderate	60

When comparing these phases with the stages of the staged model, a good match can be found. From a general inspection, as well as by reading samples of comments in commit records, the following correspondence was found. Up to 1994 (phase A), the project was in the initial development stage, producing an implementation of the C library complete enough to be adopted by major Linux distributions around that year. Phases B and C (up to 2005) both correspond to the evolution stage, although with two different activity patterns. Since 2006 (phase D), the project is in a servicing stage, characterized by the stabilization in size, and the moderate activity observed (refer to Figures 14 and 15 for the evolution in size).

**Figure 2:**
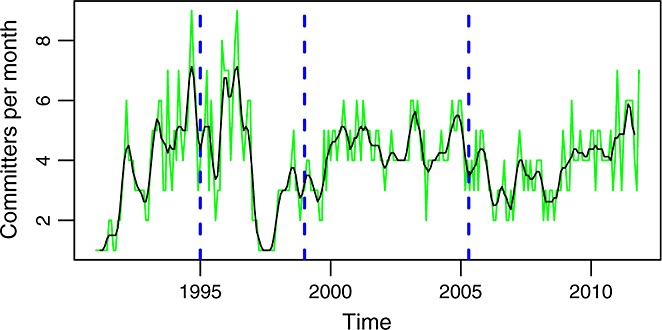
Unique committers per month. In green is the actual data. In black is a smoothed version of it (using a two-sided moving average).

With respect to the number of developers, Figure 2 shows those found active (committing) each month. The number ranges between 1 and 8 and most of the time between 2 and 6. Of course, the team of developers includes not only committers but also other people contributing in several ways, such as submitting patches. But committers, which in the case of glibc are also the core team (because active developers are promoted to committers after showing a certain level of involvement), are usually performing more than 80% of the development activity 13.

This number of developers is therefore small during all the period under study. This is so because only a few developers have been granted write access to the SCM repository. Other projects offer write permission to more developers, but glibc has always channeled contributions through a relatively small core development group. That means that the contributions coming from other people, usually in the form of patches, cannot be easily observed in the repository. In other words, for this project, we are missing the long tail of small contributors. Therefore, estimating their relevance to the whole project is something that could not be performed with our methodology.

**Figure 3:**
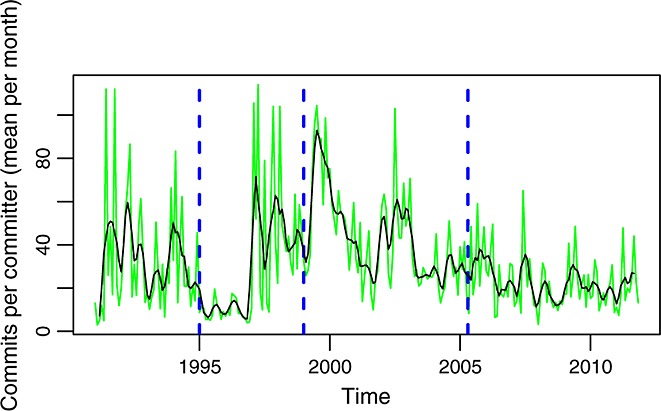
Commits per committer per month in the glibc repository. In green is the actual data. In black is a smoothed version of it.

Figure 3 shows how the ratio of commits to committers is far from constant, although some trends, compatible with those identified for commits, can be observed. In particular, the ratio is relatively high during phase A (about 30), much lower during phase B (about 10), and high again during phases C and D (although it is clearly higher during the first part of phase C).

The number of committers per month sets an upper limit to the development and maintenance effort for that month, if we neglect the contributions by those which do not have commit permission. Assuming that committers are performing almost all the work in the project during a given month, the maximum effort contributed by a team of *x* committers is *x* person-months. Of course, some of the committers could be working only part time, or even sporadically, which means the real effort spent would be lower than the upper limit. But even so, the limit may be useful as a first estimation. In the case of glibc, it is 996 person-months (83 person-years) at the end of 2011.

## 6. DEVELOPMENT ACTIVITY

The development branch is the one to be analyzed for studying evolution aspects, given that continuous growth only happens in it. Considering just one branch allows also to avoid double-counting some measurements of activity, such as counting lines added and removed over time: adding up lines added and removed only gives a meaningful result if we follow commits in a given branch.

### 6.1. Methodological issues

Some previous issues to deal with, before entering into defining the parameters of interest, are the following:

1. Can different branches be identified? If so, what happens in them? A part of the complexity of understanding the evolution of the project comes from the fact that development does not occur in a single, sequential line. On the contrary, it is common that many branches progress in parallel, passing bits of code from one to another at certain points. To complicate matters further, in FLOSS projects, it is common that many of those branches are maintained in separate repositories. To deal with this complexity, we have handled branches differently when considering global activity or ‘product activity’
  - When interested in the whole activity of the project, we have considered all branches (as in the previous section).
  - When interested in how changes were affecting the product, we focused only on the master branch.
2. Can large commits be identified and explained? One of the more prominent methodological challenges is the presence of large commits. Large commits cause discontinuities in the evolution or activity curves, making the project history more difficult to understand in those periods. In fact, large commits may be indicative for missing information in the SCM repository. The reason is that some (maybe many) changes have been combined together sharing metadata (author, time, and description, among others) instead of being committed individually with its own metadata. So, even if changes to the source code are not missing, we have lost some granularity in the changes carried out to the source code base.
  However, it is important to notice that not all large commits are due to special practices of the development team. In some cases, they are unavoidable, as well as ‘natural’, such as when the license is upgraded, and all the files have to be changed to reflect that. Fortunately enough, the combination of the activity in glibc measured in commits, changes, lines added/removed, together with comments in commit records and other methods, such as the inspection of the Changelog file, usually allow to separate both situations.

Once these issues are sorted out, we can define the parameters:

- Level of activity. As in the case of the overall activity, we use commits per month as a parameter with the same interpretation but now for the development branch.
- Amplitude of activity. We use the number of changes (each file involved in a commit is counted as a change) per month to have a complementary view to the level of activity. Although changes can be of different sizes and complexities, they are a good proxy to estimate the activity of the project. Lehman used a similar measure, the number of modules changed, added or removed, in his first work on software evolution 14.
  This parameter provides information on the amplitude of the development as well, allowing for the identification of periods that, with independence of the level of activity, have many changes to files. This is usually an indicator of perfective and preventive maintenance tasks, which are performed not in specific parts of the code (as is the case, usually, to fix a bug) but on large sets of files (such as when a rearchitecting takes place). A related measure is the number of changes per commit. This allows for the identification of outliers: large commits, which in many cases are not directly related to software development or maintenance tasks, that can distort the overall statistics for some months.
- Size of commits. The parameters of this kind count lines added and removed per month and the ratios lines added and removed per change and per commit. They allow for the identification of periods when a lot of lines are changed (be it in many different, or in a small set of files). These parameters help in the characterization according to how much the code base is changing. The same that was said for the number of files changed per commit can be said for the lines added or removed per commit: it allows for the detection of outliers.

These three views of development and maintenance activities in the development branch allow for a complete landscape of the evolution of the activity directly related to development in the project.

### 6.2 Activity in the development branch of glibc

**Figure 4:**
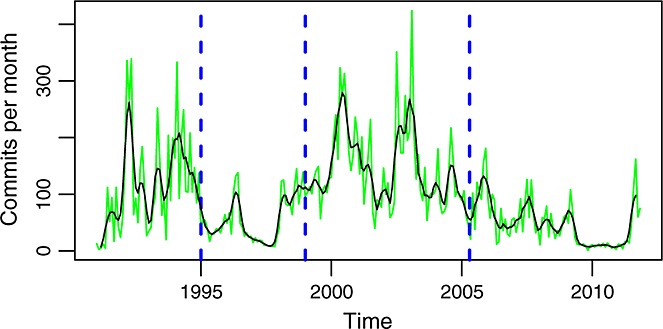
Commits per month (master branch). In green is the actual data. In black is a smoothed version of it (using a two-sided moving average).

**Figure 5:**
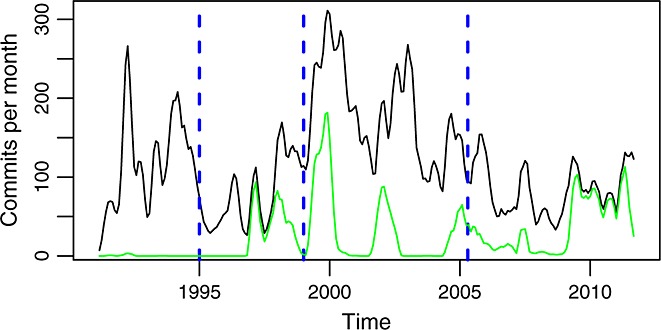
Commits per month in all the repository (black, top curve) and difference with commits per month in the master branch (green, bottom curve).

In the case of glibc, the development branch is the master branch. Figure 4 shows commits per month in it. For comparison, Figure 5 shows, for each month, the difference between all commits and commits to this branch. It can be noticed how in phase A all the activity happens in the master branch, during phase B activity in other branches starts, and runs with peaks of activity during phase C. In phase D, most of the activity has moved to other branches, coincident with the migration of the repository to git. This suggests that the new SCM system also brought new policies with respect to the work in branches, probably facilitated by the easy use of branching and merging in git.

**Figure 6:**
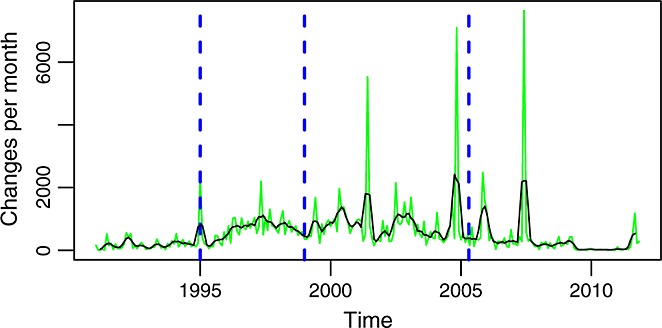
Changes per month (master branch). Changes are counted as the sum of modified files in commits.

Entering into details, Figure 6 shows the evolution over time of the number of changes. From the figure, it is clear how there are some months during which the number of changes is really high. A more detailed inspection shows how those deviations are due to extremely large commits (with a lot of files involved) happening during those months.

**Figure 7:**
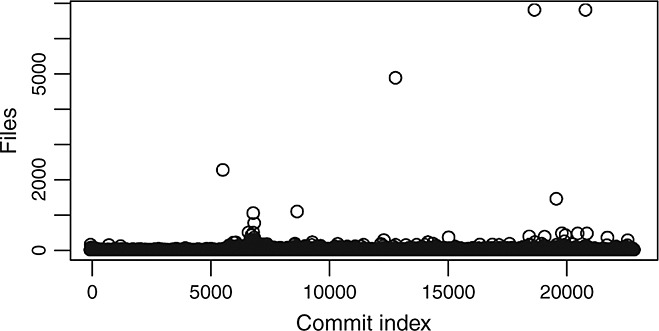
Number of files involved in each commit (commits sequentially ordered).

Figure 7 allows for their identification. It shows how most of the commits involve a relatively small number of files, except for some extreme cases. The plots for lines added and lines removed per commit show similar graphs. We calculated the percentiles of the population of commits and found that the 99% percentile involved 56 files. That is, only 1% of the top commits contain more than 56 files, and 99% of the commits involve 56 or less files. We therefore labeled all commits affecting more than 56 files as outliers.

We sorted the set of outliers (227 commits) by the number of files and analyzed the top commits in detail. Table 3 shows the top 8 commits, which affect more than 500 files each. We used the Changelog file of the project and the categories proposed by Hindle *et al*. 15 to characterize these large commits. Our results confirm that dealing with external contributions and branching and merging are the sources of these large commits. The only other case is the fourth largest commit (No. 5 in Table 3), which is due to copyright issues, as the license was changed and copyright messages had to be adapted accordingly in most files. Commit No. 1 can be considered as a merge into the repository of 2262 files that had been developed outside the SCM system.

**Table 3:** Characterization of extremely large commits.

Id	Date	Files	Lines added	Lines removed	Action/reason
1	02/18/95	2262	218,361	0	Initial import
2	06/21/97	1039	23,598	17,799	Moving and other
3	10/15/97	757	13,953	12,328	Spelling, variable names, and other
4	07/14/99	1085	43,516	38,569	Various updates
5	07/06/01	4869	40,756	40,546	Change in license
6	12/22/04	6791	337,026	526,653	Various updates
7	12/14/05	1445	39,502	45,370	Various updates
8	07/12/07	6791	526,662	337,034	Various updates

The most frequent cause for other large commits in our set corresponds to the batches of changes that were incorporated in just one commit. Using information from the Changelog file, we learned that those commits included many unrelated changes, which even correspond to different developers. This was performed that way because of the policies of access to the SCM, with committers including changes performed by others. However, only a part of the changes in the batches added functionality or changed substantial issues: most of them are changes of directory, spelling changes, or changes in the name of a variable or a macro.

**Figure 8:**
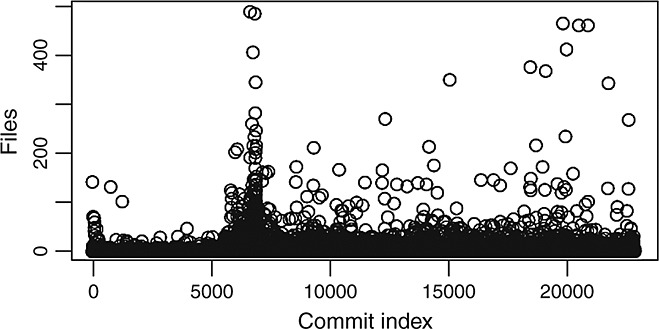
Number of files involved in each commit (commits sequentially ordered) for those commits involving 56 files or less (top 99%).

Figure 8 shows the distributions of commits size (in number of files) without including the 1% largest commits in glibc. It is clear that the distributions are now more compact. We can also observe some periodicity in the size of commits. Large commits are more frequent in certain moments of the history and are usually followed by periods with smaller commits. A more detailed inspection of those periods usually found some relationship with clean-up activities, incorporation of new code, or some rearchitecting.

**Figure 9:**
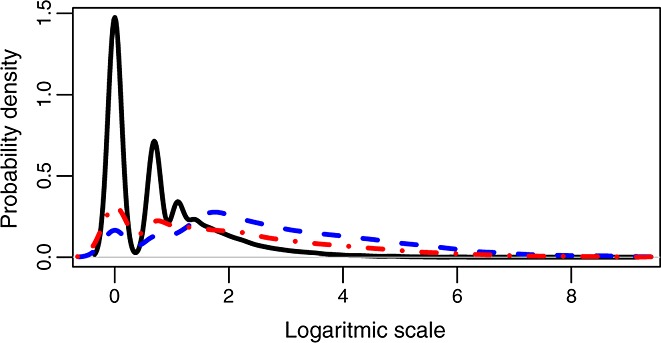
Probability distribution of the natural logarithm of the number of lines added (blue, dashed curve), lines removed (red, dash-point), and number of files (black, continuous) for each commit.

Figure 9 shows how the distribution of three parameters for each commit (lines added, lines removed, and number of files involved) is very skewed towards small numbers, although there is a tail with large commits. The peaks observed for small values correspond to the natural logarithm of the small integers. For example, for the number of files, a peak can be observed for 0 (*ln(1)*), about 0.7 (*ln(2)*), and about 1 (*ln(3)*), because there are a lot of commits involving those numbers of files. The figure shows as well that removals are smaller than additions, as the former peak at one and two lines and the latter at four lines.

**Figure 11:**
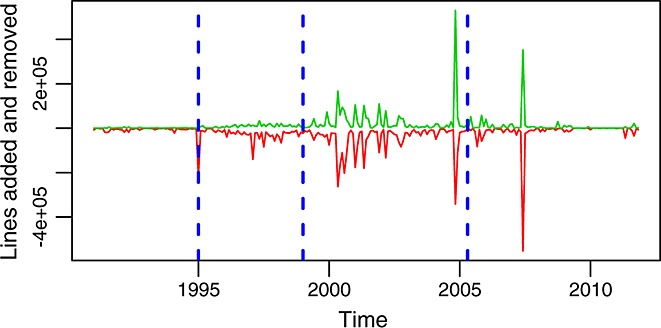
Lines added (red, bottom curve) and removed (green, top curve) per month in the glibc repository (master branch).

The shape and peaks of the time series of commits per month (Figure 4) is only in some aspects similar to those of changes per month (Figure 6). This suggests that the number of changes per commit varies a lot over time, something that can be seen in Figure 10. Not only high peaks (because of extremely large commits) can be observed but also long periods between peaks with a very low ratio of changes to commits. The comparison of Figures 11 and 6 shows a similar effect between lines added or removed for the commits in each month and the number of changes. Again, the graphs have many similarities and share some of the peaks, but they also have differences, and not all peaks are present in both. All of these graphs together mark, with their peaks, points in time where special activities took place and therefore worth studying in more detail if there is interest in reconstructing the history of the project.

**Figure:**
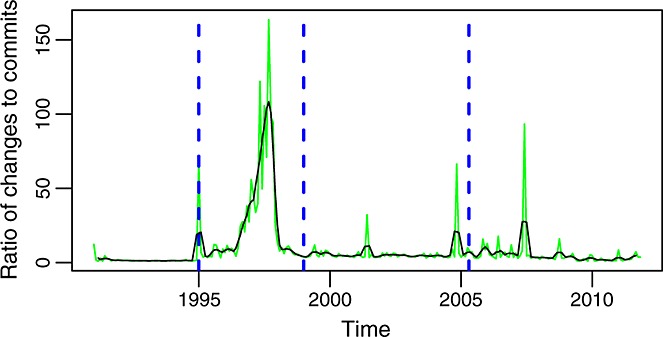
Ratio of changes to commits per month (master branch). This is also the mean number of files involved in each commit per month.

**Figure 12:**
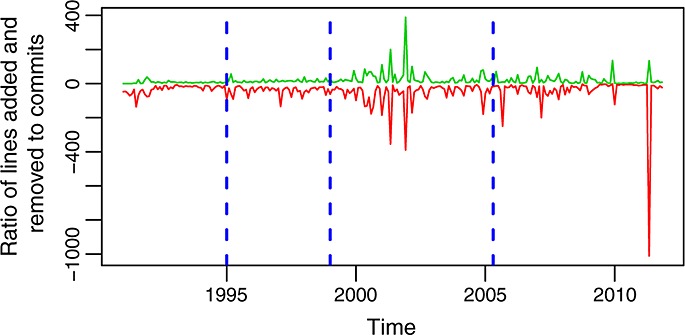
Ratio of lines added (red, bottom curve) and removed (green, top curve) to changes per month in the master branch. This is also the evolution of the mean number of lines added and removed per file.

**Figure 13:**
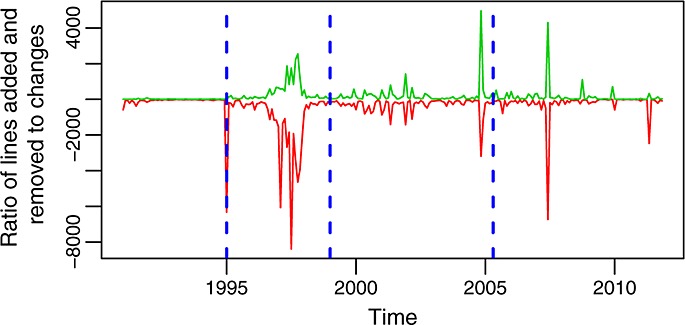
Ratio of lines added (red, bottom curve) and removed (green, top curve) to commits per month in the master branch. This is also the evolution of the mean number of lines added and removed per commit.

Figures 12 and 13 show the evolution over time of other similar ratios: lines added and removed per change and per commit. Each of these ratios tells a different part of the history of the evolution of glibc. Combining them together, it is easier to understand how the project behaved during some periods. For example, from these two latter figures, it can be better understood how during most of the life of the project individual changes are small but grow larger from 2000 to 2003, from 2005 to 2007, and in the final peak in 2011. The fact that changes are larger during those periods, all other factors being equal, suggests deeper changes, usually related to restructuring (including the incorporation or removal of large pieces of code). Another observation is the size of commits (in lines added or removed), which tends also to be small except for some short periods during which very large commits are observed. They correspond either to the incorporation into the repository of many changes in single commits or to the involvement of many files in single commits (or both). The only significant exception is the period around 1997, when the ratio of lines added and removed to commits is large during a period of several months. This is related to a long series of long commits, each of which touch small parts of many files, because of changes in names and structure in some functions, which are called from many places in the code.

## 7. SOURCE CODE EVOLUTION

After analyzing activity, which is related to the work of developers, now let us analyze the product of this work: the evolution of the source code. One of the most classic parameters to track in this case is the size of this source code over time.

### 7.1. Methodological issues

The most relevant issues in this area are the following:

- Sampling periods. Two approaches exist when doing this: evaluating growth for software releases or on a linear time scale. We have selected the latter, as the analysis of SCM repositories is more suited to this approach (we have the temporal information of all commits from the logs and commits are carried out in an almost continuous way). It is also a more reasonable approach in projects that feature a certain degree of continuous releasing, which is common in FLOSS. Additionally, we have tracked only the ‘production’ branch (master), because we are interested in understanding how this stable line of development has evolved.
- Definition of size. Size can be measured in many ways: as number of files (as traditionally performed by Lehman), as total number of lines of code (LOC, source LOC (SLOC), etc.), or even as aggregated complexity. To cover several possibilities, we have used LOC, SLOC, and number of files to measure size.

In our case, LOC are counted as ends of line (or ‘wc LOC’), SLOC are counted by using the SLOCCount tool,† and number of files include any kind of files under the control of the SCM. In all three cases, only the versions of the files in the master branch at every given time have been considered.

In addition to total SLOC, LOC, and number of files over time, we also use some other parameters:

- Comparison of LOC and SLOC. The comparison of LOC and SLOC over time provides a first impression of the amount of code, relative to non-code, in the project. In those cases when both parameters show different behaviors, it also allows for the detection of the inclusion of non-code files. Our study on glibc has shown that these changes have to be inspected with care, as an abrupt change that we at first thought was because of the addition of non-code files, finally resulted in the replacement of code files with few comments by other, more commented code files.
- LOC/SLOC per file. Given that the number of LOC/SLOC is a proxy of complexity, a study of the mean number of LOC/SLOC per file over time will give an idea of the evolution of the complexity at the file level. As the study of glibc has shown, even if the project as a whole stopped growing, the mean number of SLOC per file may increase. Both measures should be stabilized in order to enter a phase where complexity is not increasing.

### 7.2. Evolution of glibc in size

**Figure 14:**
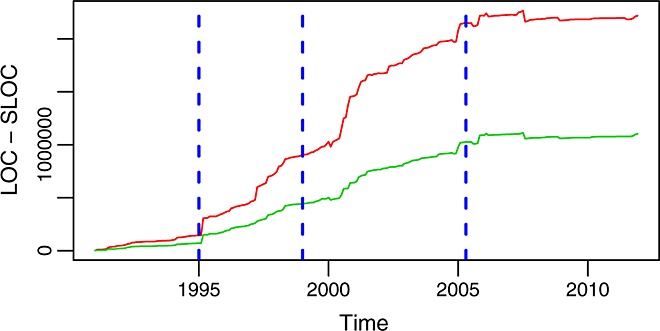
Lines of code (LOC, in red, top curve) and source lines of code (SLOC, in green, bottom curve) per month in the glibc master branch.

**Figure 15:**
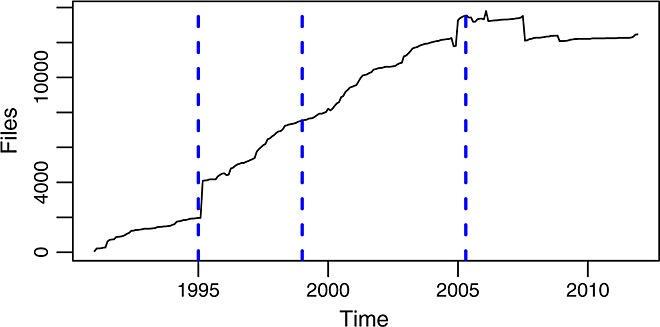
Number of files per month in the master branch.

Figures 14 and 15 show the evolution of these metrics over time. All three metrics evolve in a similar way, as it could be expected. However, when they differ, they tell interesting stories. For example, it can be observed how LOC and SLOC run quite close to each other up to 1995. At that point, LOC starts abruptly to be higher than SLOC. At the same time, the count of files almost doubles. In other words, while the lines of actual code (programming constructions) grow slowly, a lot of non-code lines and a lot of new files appear. After inspecting the source code involved, we found that this effect was because of a major refactoring. In 1995 release, 2.0 of glibc was published, and the leap corresponds to fresh code entering the repository, in many cases substituting older files that in general had fewer comments.

The evolution of the code in size, considered by itself, shows three epochs of different growth models:

- During phase A, until 1994, the code was growing at a linear, stable, and relatively slow pace. In fact, the total SLOC per month during this period can be adjusted by a line with an intercept of 5539 SLOC and a slope of 1390 SLOC/month (considering months in *x*-axis, as consecutive integers, being 0 the first month in the period) with an *R*-square of 0.9586.
- During phases B and C, from 1995 to 2005, the code goes on growing but now at a higher (although still stable) pace. SLOC per month can be adjusted for this period by a line with a slope of 7479 SLOC/month, 5.3 times larger than during phase A(the intercept being 91,137 SLOC, with an *R*-square of 0.9879).
- During phase D, the growth of the code (be it in LOC, SLOC, or number of files) stops, and the total size remains stable (between 1060 and 1110 KSLOC during all the period).

This is quite compatible with the previous observations on how during the last part of phase C commits per month were decreasing, and during phase D, they were really low. In fact, more insight can be obtained: because the size is not growing, it is likely that not much new functionality is being introduced, which means that a large fraction of the commits are due to corrective actions.

**Figure 16:**
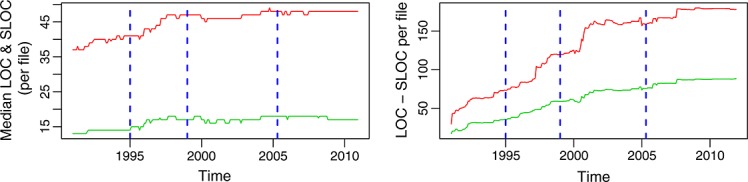
Median (left) and mean (right) of the lines of code (LOC, in red, top curve) and source lines of code (SLOC, in green, bottom curve) per file, for each month period, in the glibc repository (master branch).

Figure 16 shows another interesting fact. Although the whole product is growing in size until 2005, the median of the size of the files is very stable during phases C and D. This means that the complexity, from this point of view, was increasing slowly during phase A and a part of phase B but has been managed and kept under control since 1998.

That figure also shows how for doing this analysis, the median, and the mean tell different stories. The mean grows until around 2005 but only because a relatively small number of large files grow. The median, a much more representative parameter with such a skewed distribution of size, is also much more stable over time. This is consistent with previous studies on aggregation techniques for software metrics 16.

## 8. DISCUSSION OF THE LAWS OF SOFTWARE EVOLUTION

The combined analysis of the methodological issues identified and the data obtained in the case of glibc raise some issues worth discussing with respect to the laws of software evolution. In this section, we will start by considering the most general ones and will later deal with those affecting specific laws.

### 8.1. General issues

The main problem we have faced when exploring to which extent the laws of software evolution hold for glibc has been the difficulty in expressing them in quantitative terms that could be validated (or not) with specific measurable parameters. The laws are expressed in general terms that are both intuitive and familiar in the context of software development. This is probably needed because of the huge diversity in development environments, methodologies and processes, and therefore in parameters that could be measured in real-world cases. But this advantage becomes a problem when trying to empirically validate the laws, because it is not clear how to do that.

Literature in the field suggest some of the parameters and conditions to be used as proxies for the different concepts involved, but studies published not always agree, not always are applicable or optimal in different contexts, and in some cases, they even show diverging methodologies. As examples of this problems, refer to the previous discussions on how to measure activity (Section 6.1) or the different metrics to consider size or complexity (Section 7.1), or to the coming discussion on what is ‘constrained growth’ (Section 8.5).

This leads, unavoidably, to the core discussion of whether the laws of software evolution are falsifiable (or validable) in a clearly univocal way. That is, any two researchers, analyzing the same system, should come to the same conclusion with respect to whether it fulfills any specific law or not.

Instead of entering into this discussion, we propose to accompany the laws with specific criteria that render them validable at least for certain systems: those using SCM systems in ways similar to glibc (e.g., similar commit and development policies). Building on available literature and in our experience in the analysis of large systems such as glibc, we have selected specific parameters and conditions compatible with the statements of the laws. Those have been used to design a complete methodology that starts by the retrieval of the data in the SCM repository of a project and ends with data suitable both for the description of its evolution and for the validation of the laws.

In the rest of this section, we discuss the situation for each of the laws that we consider‡ and the definition of these accompanying criteria (parameters and conditions) that allows for their consideration with the needed precision. We only deal with some of the laws because for others (namely, laws III, VII, and VIII), we have not found parameters computable from the information in a SCM repository for expressing concepts such as ‘feedback mechanisms’ or ‘quality’, which are key to their formulation.

### 8.2. Law I, continuing change

For assessing this law, we propose three parameters, any of which would be enough to determine whether the system is being adapted or not: commits, changes (files changed), and lines added and removed (all of them per month, all of them in the development branch). Any of these parameters is a measurement of how much the system is being changed, which is a good proxy for how much the development team is trying to adapt it. If any of them shows significant activity, the other two would also show it; therefore, any of them would be enough. The only trouble could be the threshold for considering that the parameter is showing ‘continuous adaption’.

In the case of glibc, it is clear how the system is being subject to frequent changes over all its life. The evolution of the aforementioned parameters is shown in Figures 5, 6, and 10. This happens even during phase D, when no more growth is observed, and therefore, it is likely that a little new functionality is included, as the study of the comments in commit records also suggests. However, adaption to new platforms, bug fixes, and all kind of relatively small changes take place during this phase. During the other phases, this is even more clear, because the growth in size suggests an increase in functionality (which again is corroborated by the inspection of comments in commit records). Because glibc is still in use in almost all Linux-based distributions and in some other operating systems, there is no reason to assume that it is becoming less satisfactory in use (other options are available, and they have not been adopted widely).

### 8.3. Law II, increasing complexity

This law does not specify if it refers to absolute complexity (the sum of the complexity of all the components, an extensive property) or to relative complexity (the sum of the complexity of all the components relative to the number of components, an intensive property). In our case, because we use SLOC and LOC as a proxy for complexity, we focus on relative complexity, because absolute complexity would amount to absolute size of the system and is already studied in the context of law VI (refer to the succeeding texts).

Although past studies use different parameters to estimate relative complexity, we consider that SLOC and LOC are good estimators for two reasons. The first one is that when analyzed relative to components, they have a clear relation with complexity: the larger a file is, the more complex it is, all other factors equal. The second one is that there is literature showing correlation between SLOC, LOC, and some traditional complexity metrics, such as McCabe's cyclomatic complexity and Halstead's software science metrics for the case of programs written in C 17–19, and those metrics have been used in the past in the context of this law II 20. In particular, we will use the median of the SLOC and LOC per file as the parameters reflecting the relative complexity of the system.

To validate the first part of the law, a continuous increase of these parameters should be observed ‘unless work is done to maintain or reduce the complexity’. Therefore, if the project grows in relative complexity, it is clearly fulfilling the law. But if it is not, it is difficult to say anything: it could be that the project is not fulfilling the law, but it could be also that some work is performed to reduce complexity. In this latter case, other parameters should be used (such as the study of the size of changes) to find evidence of refactoring or other complexity-reducing actions.

In the case of glibc, relative complexity grows during phases A and a part of B (Figure 16, left). Therefore, the law holds during that period. From about 1998 on, the growth in complexity practically stops. Is this because of the law not holding or to activities performed to reduce complexity? The fact that changes are continuing during this time (refer to the previous subsection) would suggest that complexity-controlling tasks could be taking place. But only a more detailed analysis of the activity, probably at the level of going through each commit, would be needed to verify this.

Assessing the law requires also to study the difficulty to evolve. However, there is no evidence of an increasing difficulty. In particular, during phases A and B, the system is growing at different paces, but the core developer team is not increased in proportion. In fact, the stabilization on relative complexity during phases C and D suggests that, if more difficulties were found, the project is capable of managing them well enough to maintain it.

### 8.4. Law IV, conservation of organizational stability

For assessing the validity of this law, we would need data about the total effort put into the development, which cannot be found in the SCM repository. However, the SCM repository does contain information about an activity related to changes in the source code, which can be considered as a proxy for effort. Therefore, we propose commits per month as the parameter to use in this case.

The use of this parameter is well supported in the literature. It can be tracked back to 13, one of the first works comparing FLOSS and non-FLOSS project, which already considered commits as ‘modification requests’. Although there are other activities that do not leave traces or leave little traces in the SCM repository, modification requests, a well-known unit of activity in the industry, only takes into account work that translates into changes to the code base of the project. For some of those other activities, such as changes performed outside the SCM and latter committed in large commits with many of those changes each, at least there are some traces in the SCM repository. But for others, such as discussions in mailing lists, nothing is perceived in it. Therefore, the parameter we propose could be considered as a lower bound for activity, and it will be more or less representative depending on how much of it happens in other places.

For glibc, this law seems to hold to a certain extent. Figure 1 shows how for each of the periods, the activity is somewhat stable. However, there are many spikes in the number of commits per month, and commits have different sizes. Because the law is not very specific in what ‘tends to be constant’ means, it is difficult to say if it holds or not. This said, it is clear that the evolution in size is not linked to the activity in the repository. That would suggest that even if the activity is not completely constant, it is certainly ‘stable’, in the sense that it does not depend on the characteristics (such as size) of the product.

If the activity outside the core development team were taken into account, maybe this law would not hold, because it seems there is a relationship between the popularity of the product and the effort available for it, as the transition from phase A (when glibc was not used in any popular operating system) to phase B (when became standard in Linux-based distributions) suggests.

### 8.5. Law V, conservation of familiarity

Of the many senses in which a system may be considered to grow, we propose size as the parameter to track for this law, in line with most evolution studies. As in the case of the second law, both total LOC and total SLOC per month can be used.

But this law presents a specific ambiguity: ‘constrained’ is not strictly defined, which renders any quantitative analysis difficult. At least two widespread proposals of which growth patterns are considered to be non-constrained growth can be considered: Israeli and Feitelson 20 considered linear growth as such, whereas Godfrey and Tu 21, when analyzing the Linux kernel, considered (and found in that case) superlinear growth. All interpretations seem to agree on sublinear growth as a clear case of constrained growth.

In the case of glibc, it was already shown how the growth during phases A, B, and C follows a clear linear pattern (although at very different paces, refer to Section 7). Can this growth be considered as constrained? Probably that depends with the comparison, or with what could be considered ‘a project running at its full, unconstrained potential’. Following Godfrey and Tu, it could be said that the project is not capable of reaching superlinear growth, which means some kind of constraints (because of the need of maintaining familiarity or to other reasons) are in place. However, it can also be said, more in line with Israeli and Feitelson, that the project is able of sustaining linear growth during long periods.

Maybe the key (which is probably the key in the discrepancy between these two views of what does ‘unconstrained’ mean) lies in the team of developers. During most of the considered period, the team of committers is of six persons of less (Figure 2). This certainly puts a limit on growth: without an expanding development team, superlinear growth cannot be maintained, because there is a limit on the activity that a limited team can sustain. From this point of view, glibc behaves according to what is expected in traditional software development with stable development teams. The Linux kernel and other projects have found ways of expanding the developer base without being constrained by the need to maintain familiarity, by using hierarchical development and modularity, thus being able of sustaining superlinear growth. The glibc behaves and it can, given its limited core development team: maintaining linear growth.

During phase D, there is no further growth, which implies even a greater constraint. However, the reason is more likely to be the project entering into a servicing stage than the need to maintain familiarity what limits growth in this case, because the same need allowed for linear growth in the previous phases. This is the only phase when sublinearity is found in the growth of the source code.

### 8.6. Law VI, continuing growth

In this case, a specific measure of functionality (such as function points) should be used. But again, that parameter cannot be extracted from the SCM repository. Therefore, we propose size as a proxy for it: if the size of the source code for a program grows, it is likely that its functionality is also growing and vice versa. This has been used in the past in the literature, including the original works by Lehman 9. Again, size can be expressed in SLOC or LOC per month.

It was already discussed how size has been growing during phases A, B, and C, which would mean the law holds. But it would not hold during phase D, when there is no more growth. However, if we consider the staged model for software evolution, introduced in Section 5, this could be because of the project entering the servicing stage. In this stage, no growth is observed because almost the only activity is fixing problems, adding little or no new functionality. In the context of the sixth law, it would imply that the library is starting to loss adaption to new situations and would eventually fail to satisfy the needs of an increasing number of users.

## 9. THREATS TO VALIDITY

This study presents several threats to validity. To discuss them, we use the usual distinction between threats to internal (the study itself) and external (extension to other cases) validity.

### 9.1. Threats to internal validity

Because one of the main objectives of the study is to decide on the validity of the laws of software evolution in the case of glibc, the first point to deal with is if glibc is an *E*-type system. If it were not an *E*-type, laws would not be applicable, and nothing relevant could be said. This is therefore a very important issue, and as such, it was already discussed in Section 3.1. We expect that reasonable doubts were addressed there, but of course, if some doubt about that still holds, the result could be completely void from that point of view.

Going down in order of importance, the next threat is to which extent the metrics extracted from the SCM repository are good proxies for the parameters mentioned in the laws of evolution and for the other conclusions presented in the previous discussion. This has been extensively discussed in the previous section.

Another threat to our study is the absence of the complete development information. So, projects may import code that seems to come out of the blue but had its own development process and its own evolution. Historically, this has been recognized as a way of fighting the complexity of the real world: problems are solved elsewhere and incorporated (when the code for complete modules is reused) to the repository or specific needs are addressed in those parallel lines and later merged. However, positive from the point of view of adaption to new circumstances, this has also the negative aspect of needing a lot of effort in the merging process, which translates into continuous merging activities during all the life of the project. Although modern SCM systems resolve merging and branching better, in the case of older projects, this may cause some distortion.

There are specific threats that can be identified in the process of retrieving data from the SCM repository and its analysis, such as the identification of snapshots of code at given points in time, and identification of the complete list of actions related to branches. These are complex tasks and therefore error-prone. However, we have automated our information recovery process using CVSAnalY2, a tool that is built and maintained in our research team, which is being used by many other researchers and which has gone, therefore, through several testing and verification activities in studies recovering information from control version repositories.

Finally, the SCM repository itself could contain errors. In fact, we have identified some of them, which were already explained, such as the date of the first commit. In particular, the migration from CVS to git and the first imports of files into CVS are prone to include errors. In addition, the presence of large commits corresponding to packs of changes makes it also difficult to produce in some cases meaningful metrics.

### 9.2. Threats to external validity

The most obvious threat is that the methodology and study have only been performed on a single project. This could render any specific result of its application to glibc, such the validity of a given law, as invalid in any other case. However, the methodology is general enough to be useful for the analysis of any project using the SCM system in a way similar to glibc. In this respect, there are some issues worth mentioning:

- Different periods of study. Months are a compromise between having periods large enough to aggregate information and short enough to not mask short-term patterns. In principle, other periods could show different evolution patterns and should be explored in specific cases. The use of a two-sided moving average smoothing, shown in the graphs (we used a left and right 2 months smoothing windows), may help to perceive long-term trends. For glibc, we have repeated the study with weeks and quarters as the periods, with very similar results.
- Identification of multiple identities of developers during the periods of study. Because the methodology assumes that the activity of developers is studied, it is important to match identities to real developers: otherwise, developers would be overcounted. Fortunately, it is not that common that a developer uses multiple identities during the same period.
- Lines as size of commits. Depending on the programming language, on whether a file has a source code or text, etc., the size of commits with similar ‘importance’ may be different. In the case of our methodology, this would usually have little impact on the long-term trends, but in specific cases of mixtures of very different programming languages or of special relevance of files not with a source code, this effect should be explored.

This leads to another threat: to which extent projects use SCM repositories in ways that make them comparable. We have already seen how the transition from CVS to git has had an impact in the behavior of the project, which raises some doubts of the usefulness of comparing results among projects with different kinds of SCM systems. But even for the same kind of SCM, it could be used in different ways. We have seen how large commits reflect practices of developing elsewhere, and the commit all changes at a specific point in time. This kind of practices could be very specific to each project, again rendering comparisons not useful at all.

And of course, the whole methodology used assumes that the product is developed and maintained through interactions with the SCM system. If the project uses no SCM, we used it in very different ways (such as for maintaining backups of the real development tree or just for coordination between different development repositories), most of the conclusions would again be void.

## 10. PREVIOUS RESEARCH

Since the publication of Lehman's seminal paper 22 that started the field of software evolution, many systems have been analyzed from this point of view 23. The laws of software evolution have evolved themselves since their first formulation until their last formulation 1, to adapt to the new lessons learned from this analysis.

However, during the last decade, the public availability of the development repositories of many mature FLOSS projects has meaningfully lowered the barriers for the data-based, empirical analysis of software projects from many points of view 24,25. This has had an impact on the software evolution field, because those repositories usually include the kind of information needed to perform evolutionary analysis. In fact, one of the earlier studies on FLOSS projects was a paper by Godfrey and Tu 26 (later confirmed, at least in part, in other studies 27,20) showing how the Linux kernel was growing at a superlinear pace, in apparent contradiction with the prediction, on the basis of the laws of software evolution, that the growth of software projects must slow down with time because of the need to handle increasing complexity. Lehman considered this case 28, signaling some methodological issues to deny its validity to test the laws.

The number of studies seeded by Godfrey and Tu grew during the decade of the 2000s, including large-scale studies 29 and meta-studies 30 focused on studying the validity of the laws for FLOSS projects. In addition to the interest that FLOSS development models raise, this was possible because of the already mentioned large-scale public availability of detailed data about software development that most FLOSS projects provide. All these developments have led to the publication of two books 31,23 that explore the diversity of the published results to conclude that it is not clear that the laws have been invalidated, at least to a large extent. But it is also difficult to argue the contrary. What is clear at this point is that only methodological advances, clear consensus on the metrics to consider and the exact meaning of some of the laws, and statistical analysis of very large sets of screened projects (to avoid those too young, too little used, too small, etc.) can help to reach a further scientific evidence about the validity of the laws.

The measures proposed in this study are backed by previous work, but we have to recognize that the lack of consensus on which metrics are worthy makes it difficult to select them. For example, LOC has been denied by Lehman *et al*. 32 as a good metric to measure size, when compared with other higher level metrics such as SLOC. However, they have been shown to be comparable in several cases 19.

The already mentioned study by Israeli and Feitelson 20 was the first one providing an extensive quantification of the laws, using several metrics for size: LOC, number of system calls, number of functions, and so on. We have used similar parameters for size.

With respect to the data sources, traditional studies of software evolution are based on the analysis of software releases. Our study, on the contrary, can be included in a line of research that uses not only the state at release time (the ‘released product’) but also the whole story of development (the ‘developed product’) as a source of data for the study 33,29. This approach is not necessarily better than the other but is certainly different. In our case, all the artifacts needed for the development of the product are considered, including tests, configuration files, and so on. On the other hand, only delivered code, which usually includes only the software, are intended to be run by end users. The timing approach is also different: we consider almost continuous change, whereas the other approach is discreet, considering only snapshots at release times.

## 11. CONCLUSIONS

To our knowledge, this is the first study of 20 years of the life of a software project, on the basis of information from its SCM repository, with the combined view produced by the study of the general activity of the developers, of the particular activity in the production branch of development, and of the evolution of the size of the code. We have been able to do it thanks to the availability of data for all this period in the current glibc git repository and to its public availability. When looking for long-lived FLOSS projects, we found that it is not common to have all their history available in their SCM repository or that it had problems (such as periods without information) that rendered them unusable for a study such as this one.

We have been able to describe the history of glibc to a large detail showing its activity and evolution patterns over time and finding some reasons to the findings. In particular, we have identified four phases in the development of the library and have related them to external events, such as the use of glibc by most major Linux-based distributions.

The study was designed from the beginning for describing the history of glibc in the context of the laws of software evolution. In this respect, we have analyzed several of the laws, comparing their statements to the data in our case study. We have found several cases in which the laws do not seem to hold and a few others where they do. In particular, we have found that the first law clearly holds, the second law holds only in part and during some periods, the fourth law holds at least to a certain extent, the fifth law does not seem to hold during most of the life of glibc, and the sixth law does not hold during the last phase of the project.

For doing this validation of the laws, we also proposed a set of parameters that complement the relatively vague statements of the laws. Those parameters are all computable from the information in a SCM repository. We have proposed also a repeatable methodology that, starting from the SCM repository, produces the parameters used for the description of the evolution of the project and the assessment on the validity of the laws.

The study has also produced some other side outcomes. We have presented not only a brief introduction to a model of extended evolution, including branches in the official SCM repository of a project, but also code developed elsewhere and have discussed its implications for the field of software evolution and the management of complexity. We have also performed an identification and analysis of the extremely large commits that appear in the glibc git repository.

From a more methodological point of view, the paper shows in detail which information related to software evolution can be obtained from a SCM repository and the problems found with a real case when this methodology is to be used in large, long-lived projects. In the companion web site with reproducibility elements (refer to the succeeding texts), all the practical details leading from the retrieval of information from git to the production of the graphs shown in the paper are provided, filling all the gaps needed to understand how a study of this kind and scale can be performed and providing the necessary software for it.

One specific side result is related to the problems we have had to classify glibc in the SPE schema. Maybe this is pointing to operational problems on the application of that schema to classify some software systems.

In summary, although this paper does not pretend to show results that are general to other projects, it does provide details on a methodology that could be applied, with no or little change in other cases, with the aim of understanding how large, long-lived software projects evolve over time.

Many future lines of research are now possible. Among them, performing similar studies on large quantities of projects, to gain statistical evidence of how they evolve, remains an open issue. This paper intends to give a step further, by providing the first steps for a methodology that could be used in that large scale. But the methodology itself needs to be refined and adapted to other kinds of SCM systems and be tailored to produce not only graphs but also parameters that describe the evolution of a project in a way that can be used to validate (or not) and to complement the laws of software evolution and to compare the results of large samples of projects. In addition, considering information from other kinds of repositories, such as issue tracking systems, would be a good complement to better understand evolution. On a complementary line, performing deep, detailed studies of more large projects will help to improve our knowledge of the issues involved in this area and to better produce the tools needed for the large-scale studies formerly mentioned.

## 12. REPRODUCIBILITY ELEMENTS AND METHODOLOGICAL DETAILS

This study has been performed by using the following tools: CVSAnalY2, MySQL, and R. The data source used has been the official git repository of the glibc project. The exact versions of the tools and data source used, the details and scripts used to process and to analyze the information, and other complementary information can be found in the companion web site with reproducibility elements and methodological details (http://gsyc.urjc.es/∼jgb/repro/2012-jsme-long-evolution), built following the criteria presented in 34.

## Footnotes

* CVSAnalY2 is a part of the Metrics Grimoire, a toolset for retrieving information from software development repositories http://metricsgrimoire.github.com/.

† SLOCCount, by David Wheeler, http://www.dwheeler.com/sloccount/.

## References

[1] Lehman MM (1996). Laws of software evolution revisited. Proceedings of the European Workshop on Software Process Technology.

[2] Belady LA, Lehman MM (1971). >Programming system dynamics or the metadynamics of systems in maintenance and growth. Research Report RC3546, IBM.

[3] Lehman MM (1980). Programs, life cycles, and laws of software evolution. Proceedings of the IEEE.

[4] Cook S, Harrison R, Lehman MM, Wernick P (2006). Evolution in software systems: foundations of the SPE classification scheme. Journal of Software Maintenance and Evolution: Research and Practice.

[5] Free Software Foundation (1988). GNU's bulletin, vol. no. 4 Feb.. http://www.gnu.org/bulletins/bull4.html.

[6] Free Software Foundation (1992). GNU's bulletin, vol. no. 12 Jan.. http://www.gnu.org/bulletins/bull12.html.

[7] Bird C, Rigby PC, Barr ET, Hamilton DJ, Germn DM, Devanbu PT (2009). The promises and perils of mining git. Working Conference on Mining Software Repositories 2009.

[8] Feitelson DG (2012). Perpetual development: a model of the Linux kernel life cycle. Journal of Systems and Software.

[9] Lehman MM, Belady LA (1985). Program Evolution. Processes of Software Change.

[10] Rajlich V, Bennett K (2000). A staged model for the software life cycle. IEEE Computer.

[11] Capiluppi A, González-Barahona JM, Herraiz I, Robles G (2007). Adapting the ‘staged model for software evolution’ to free/libre/open source software.. IWPSE'07: Ninth international workshop on Principles of software evolution.

[12] Capiluppi A, Michlmayr M, Feller J, Fitzgerald B, Scacchi W, Sillitti A (2007). From the cathedral to the bazaar: an empirical study of the lifecycle of volunteer community projects. Open Source Development, Adoption and Innovation IFIP International Federation for Information Processing.

[13] Mockus A, Fielding RT, Herbsleb JD (2002). Two case studies of open source software development: Apache and Mozilla. ACM Transactions on Software Engineering and Methodology.

[14] Lehman MM, Lehman MM, Belady LA (1985). The programming process. Program Evolution. Processes of Software Change.

[15] Hindle A, German DM, Holt R (2008). What do large commits tell us? a taxonomical study of large commits.. Proceedings of the 2008 International Working Conference on Mining Software Repositories.

[16] Vasilescu B, Serebrenik A, Van Den Brand M (2011). You can't control the unfamiliar: a study on the relations between aggregation techniques for software metrics. Software Maintenance (ICSM), 2011 27th IEEE International Conference on.

[17] Graves TL, Karr AF, Marron J, Siy H (2000). Predicting fault incidence using software change history. IEEE Transactions on Software Engineering.

[18] Herraiz I, Hassan AE (2010). Beyond lines of code: do we need more complexity metrics? O’ Reilly Media,. Making software, chap..

[19] Herraiz I (2009). A statistical examination of the evolution and properties of libre software.. Proceedings of the 25th IEEE International Conference on Software Maintenance (ICSM).

[20] Israeli A, Feitelson DG (2010). The Linux kernel as a case study in software evolution. Journal of Systems and Software.

[21] Lehman MM (1974).

[22] Mens T, Demeyer S (2008). Software Evolution.

[23] Crowston K, Wei K, Howison J, Wiggins A (2011). Free/libre open source software development: what we know and what we do not know. ACM Computing Surveys.

[24] Androutsellis-Theotokis S, Spinellis D, Kechagia M, Gousios G (2010). Open source software: a survey from 10,000 feet. Foundations and Trends in Technology, Information and Operations Management.

[25] Godfrey M, Tu Q (2001). Growth, evolution, and structural change in open source software. Proceedings of the International Workshop on Principles of Software Evolution.

[26] Godfrey MW, Tu Q (2000). Evolution in open source software: a case study.. Proceedings of the International Conference on Software Maintenance.

[27] Robles G, Amor JJ, Gonzalez-Barahona JM, Herraiz I (2005). Evolution and growth in large libre software projects.. Proceedings of the International Workshop on Principles in Software Evolution.

[28] Lehman MM, Ramil JF, Sandler U (2001). An approach to modelling long-term growth trends in software systems,. Internation Conference on Software Maintenance.

[29] Koch S (2007). Software evolution in open source projects—a large-scale investigation. Journal of Software Maintenance and Evolution: Research and Practice.

[30] Fernandez-Ramil J, Lozano A, Wermelinger M, Capiluppi A, Mens T, Demeyer S (2008). Empirical studies of open source evolution. Software Evolution.

[31] Madhavji NH, Fernandez-Ramil J, Perry DE (2006). Software Evolution and Feedback. Theory and Practice.

[32] Lehman MM, Perry DE, Ramil JF (1998). Implications of evolution metrics on software maintenance.. Proceedings of International Conference on Software Maintenance.

[33] Herraiz I, Gonzalez-Barahona JM, Robles G (2007). >Towards a theoretical model for software growth,. International Workshop on Mining Software Repositories.

[34] Gonzalez-Barahona JM, Robles G (2012). On the reproducibility of empirical software engineering studies based on data retrieved from development repositories. Empirical Software Engineering.

